# Blame Conformity: Innocent Bystanders Can Be Blamed for a Crime as a Result of Misinformation from a Young, but Not Elderly, Adult Co-Witness

**DOI:** 10.1371/journal.pone.0134739

**Published:** 2015-07-31

**Authors:** Craig Thorley

**Affiliations:** Department of Psychological Sciences, University of Liverpool, Liverpool, England; University of Calgary, CANADA

## Abstract

This study examined whether or not exposing an eyewitness to a co-witness statement that incorrectly blames an innocent bystander for a crime can increase the likelihood of the eyewitness subsequently blaming the innocent bystander for the crime. It also examined whether or not the perceived age of the co-witness influences this effect. Participant eyewitnesses first watched a video of a crime featuring a perpetrator and an innocent bystander. They then read one of six bogus co-witness statements about the crime. All were presented as having been written by a female co-witness and they differed in terms of her age (young adult or elderly) and who she blamed for the crime (the perpetrator, the innocent bystander, or nobody). One week later the participants were asked who committed the crime. When the young adult co-witness had blamed the innocent bystander just over 40% of participants subsequently did the same. Few participants (less than 8%) in the other conditions subsequently blamed the innocent bystander. The elderly co-witness was also rated as less credible, less competent, and less accurate than the younger co-witness suggesting eyewitnesses were less likely to be influenced by her incorrect statement as they perceived her to be a less reliable source of information. The applied implications of these findings are discussed.

## Introduction

There are hundreds of documented legal cases where innocent people have been accused, tried, convicted, imprisoned, and sometimes executed, for crimes they did not commit [1–3]. Wrongful convictions can occur for a number of reasons including false confessions and improper forensic analysis. In the United States, however, 76% of the first 250 people exonerated of crimes as a result of DNA evidence were convicted, at least in part, as a result of mistaken eyewitness identification [4]. A large literature exists examining the reasons why mistaken eyewitness identifications occur (for an overview see [5]). A small number of studies within this literature have demonstrated that eyewitnesses can sometimes mistakenly identify an innocent bystander, visible during a crime but not directly involved in it, as the perpetrator (e.g., [6]). This study examines whether or not eyewitnesses are at greater risk of blaming an innocent bystander for a crime if they are exposed to post-event misinformation in a co-witness statement that suggests the innocent bystander was the perpetrator. This study also examines whether or not the age of the co-witness who wrote this statement impacts upon the effect. Here, the co-witness was either a young adult or elderly.

Eyewitnesses can spontaneously mistakenly identify an innocent bystander as the perpetrator of a crime (or other incident). This effect is known as *unconscious transference* [7–8]. Buckhout [6] demonstrated this in a study where 141 unsuspecting students witnessed a staged assault on a professor by a stranger. Throughout the assault an innocent bystander was stood nearby. Seven weeks later the students had to identify the perpetrator from a six person photo lineup. One of the photographs was of the innocent bystander. Only 40% of the students correctly identified the perpetrator. A further 25% mistakenly identified the innocent bystander as the perpetrator. Ross, Ceci, Dunning, and Toglia [9] suggest that unconscious transference can occur for two reasons. In some instances eyewitnesses will have no conscious recollection of seeing an innocent bystander during a crime but information about this person will have been implicitly learned and stored in memory. When the eyewitnesses subsequently see the innocent bystander in a lineup, this person feels familiar. As eyewitnesses would only expect a feeling of familiarity upon seeing the perpetrator, the innocent bystander is assumed to be the perpetrator. In other instances eyewitnesses will consciously recollect seeing the innocent bystander and the perpetrator at the crime scene and create a separate memory for each. During a lineup, however, the two memories become confused and the innocent bystander is identified as the perpetrator.

It is established that if eyewitnesses encounter post-event misinformation that incorrectly describes crime details then this same misinformation can taint the eyewitnesses’ subsequent recollection of the crime (for reviews see [10–11]). One source of post-event misinformation is leading questions which eyewitnesses can encounter when questioned about a crime by police officers, lawyers, friends, or others [12]. A literature search suggests that only one study has investigated whether or not leading questions implying an innocent bystander was to blame for a crime can increase the likelihood of this person being mistakenly identified as the perpetrator. In this study, Miller and Loftus [13] had participants read a story about an argument amongst six students that culminated in an assault. Accompanying the story were pictures of each student, one of whom was an innocent bystander wearing a hat. Immediately after the story half of the participants were asked a leading question by the researcher that implied the person in the hat had committed the assault. Three days later the participants were shown pictures of the six students and had to identify the attacker. 24% of participants exposed to the leading question mistakenly identified the innocent bystander in the hat as the attacker, compared to only 6% of participants not exposed to the leading question.

The above research shows three different ways in which an innocent bystander can be mistakenly identified as the perpetrator of a crime. At this juncture it is important point out two key differences that exist between this earlier work and the current study. First, the current study exposes participant eyewitnesses to post-event misinformation about who was to blame for the crime via a co-witness statement (and not a leading question). Second, participants will be questioned about who committed the crime (and will not complete a lineup). Both changes are forensically relevant. The first change is forensically relevant as most crimes have multiple eyewitnesses and information about the crime can be shared between these co-witnesses throughout a criminal investigation [14–15]. This co-witness information can be shared ‘directly’ during discussions or ‘indirectly’ through a third party (e.g., a police officer) who informs one eyewitness about what another said [16]. There are several documented real world cases where eyewitnesses have been exposed to co-witness misinformation and this same misinformation has been incorporated into their subsequent legal testimonies (for examples see [17–19]). When one individual alters their memory report of an event to be consistent with another’s differing memory report of the same event this is known as *memory conformity* [20] or social contagion of memory [21]. It is therefore important to determine whether or not exposing eyewitnesses to co-witness misinformation that suggests an innocent bystander was the perpetrator of a crime can increase the risk of the eyewitnesses later blaming the innocent bystander for the crime. The second change is forensically relevant as eyewitnesses are often questioned by the police about a crime in the initial stages of an investigation. Some of these questions will focus upon who the perpetrator was and a description will be taken. Lineups typically only take place after this initial questioning. It is therefore also important to determine whether or not eyewitnesses who are exposed to co-witness misinformation that blames an innocent bystander for a crime are at increased risk of subsequently blaming the innocent bystander for the crime when their memory of it is assessed in this way.

A number of different techniques can be used to directly or indirectly expose participants to co-witness misinformation during laboratory studies and induce memory conformity. A popular method of direct exposure involves having participant and confederate pairs study and remember the same information together. During the collaborative remembering test the confederate deliberately claims to remember non-studied information. On subsequent individual testing the participant often succumbs to memory conformity by also claiming to remember the same non-studied information (e.g., [21–28]). A popular method of indirect exposure is to have individual participants study some information, then have them read or listen to a hypothetical co-participant’s erroneous recall of this same information, and then have them complete an individual memory test. Again, participants often succumb to memory conformity and claim to remember the non-studied information (e.g., [23–25; 29–32]).

It has yet to be determined whether or not exposing eyewitnesses to co-witness misinformation that blames an innocent bystander for a crime can increase the risk of the eyewitness subsequently also blaming the innocent bystander for the crime. Thorley and Rushton-Woods [33], however, provide evidence to suggest that attributions of blame for an incident can be shifted between individuals as a result of indirectly encountered co-witness misinformation. In their study, participant eyewitnesses watched a video of an accident involving two men, both of whom were equally responsible for its occurrence. Participants then read one of three co-witness statements about the accident that differed in terms of who the co-witness blamed (Man 1, Man 2, or blame not mentioned). When no blame was mentioned in the statement less than 2% of participants subsequently attributed blame to one of the two men. When the co-witness statement blamed one of the men for the accident 37% of participants also blamed this same man. Thorley and Rushton-Woods referred to this specific type of memory conformity as *blame conformity* to try and capture the fact that participants shifted their attributions of blame to be consistent with those of a co-witness. Thorley and Rushton-Woods observations cannot directly address the question as to whether or not co-witness post-event misinformation can lead to innocent bystanders being blamed for a crime. They do, however, demonstrate that attributions of blame can be influenced by this form of post-event misinformation and suggest such an effect may be possible.

The age of a co-witness (or co-participant) imparting post-event misinformation is an important factor in determining whether or not a person succumbs to memory conformity. This has been studied when the misinformation is encountered both directly and indirectly. With respect to the former, Davis and Meade [34] demonstrated that young adult and elderly participants will succumb to memory conformity when a confederate is a young adult but neither succumbs to memory conformity when a confederate is elderly. With respect to the latter, Kwong See, Hoffman, and Wood [35] found that young adults conformed to misinformation in a co-witness narrative when it supposedly derived from a young adult co-witness but not an elderly co-witness. Kwong See et al. [35] also asked participants to rate the co-witness on a range of dimensions including competence. Despite the fact that the narratives from both co-witnesses were identical, the participants rated the elderly co-witness as less competent and this rating predicted the degree to which they succumbed to memory conformity. Given these findings it seems likely that a participant will be more likely to blame an innocent bystander for a crime after being exposed to co-witness misinformation from a young adult, compared to an elderly adult, that blames the innocent bystander for the crime.

The first aim of this study was to examine whether or not participant eyewitnesses will engage in blame conformity with a co-witness who suggests that an innocent bystander was the perpetrator of a crime. As in many previous studies on co-witness misinformation effects, this misinformation will be delivered indirectly in the form a co-witness narrative (e.g., [35]). In this narrative blame for the crime will be placed on the perpetrator, an innocent bystander, or no blame will be mentioned. The second aim of this study was to examine whether or not differences in the age of the co-witness who supposedly wrote the narrative impacts upon blame conformity. This will be explored by informing participants that the co-witness was either a 21 year old or an 82 year old female. Based on past research demonstrating that eyewitnesses will engage in memory conformity/blame conformity with a young adult co-witness, it was anticipated that participants who read an incorrect statement from a young adult co-witness will be at greater risk of blaming the innocent bystander for the crime than those who read a correct statement from this person or a statement in which she does not mention blame. Based on past research demonstrating that eyewitnesses are less likely to engage in memory conformity with an elderly co-witness, it was anticipated that participants who read an incorrect statement from an elderly co-witness would be at no greater risk of blaming the innocent bystander for the crime than those who read a correct statement from this person or a statement in which she does not mention blame. Finally, participants will also be asked to rate the co-witness on five characteristics (accuracy, confidence, honesty, credibility, and competence). Consistent with past research by Kwong See et al. [35] it is anticipated that participants will rate the elderly co-witness as less competent than the young adult co-witness.

## Method

### Participants

The sample consisted of 168 adults (111 females, 57 males) aged 18–70 (*M* = 21.45, *SD* = 7.32). They were recruited via advertisements placed across the University of Liverpool. Participants were a combination of staff and students. Participants received either course credit or a small payment for participation. The study protocol was approved by the University of Liverpool Institutional Research Ethics Committee and was conducted according to the ethical standards laid down in the Declaration of Helsinki (1964). All participants gave informed signed consent prior to taking part.

### Design

The study had a 3 x 2 between-subjects design with 28 participants randomly allocated to each of the six conditions. The first factor was co-witness statement type. This varied according to whether the co-witness blamed no one for the crime, correctly blamed the perpetrator for the crime, or incorrectly blamed an innocent bystander for the crime. The second factor was co-witness age, with her being either a young adult (21 years old) or elderly (82 years old). The dependent measures are described next.

### Stimuli and Dependent Measures

Six stimuli were used in this study: (i) a crime video, (ii) a filler video (iii) a picture of a co-witness who observed the crime, (iv) a statement from the co-witness about the crime, (v) a 10-item multiple choice recognition test about events in the crime video, and (vi) a 6-item questionnaire about the co-witness.

The crime video centres on a young adult male selling a stolen camera in a pub to a young adult female. It was professionally filmed for police training and lasts 1 min 11s. It begins with the camera seller entering the pub. He approaches two different sets of customers who are sitting at tables and asks them if they would like to buy a camera. On both occasions his offer is turned down. In the critical scene the camera seller approaches a third table where three young adults in their early twenties are sitting together having a drink. The group consists of one male with short brown hair who is wearing a light brown coloured shirt, one female with short (above the shoulder) dark brown hair who is wearing a white fleece with a zip-up collar, and one female with long (past the shoulder) light brown hair who is wearing a grey jumper. The two females are therefore easily distinguishable in terms of their hair colour, hair length, style of clothing, and colour of clothing. The camera seller asks the group if they would be interested in buying a camera and the female with the short dark brown hair and white fleece expresses an interest. The camera seller sits at the table, passes her the camera under the table to look at, they agree on a price for the camera, and she passes him the money under the table. The camera seller then leaves the group and the video ends. Throughout the video only a small section of the pub is shown and background noise suggests that other people are in the venue at the time of the incident but that they are out of camera shot. The crime video depicts two criminal offences under local English and Welsh law [36]. First, it is illegal to sell stolen property. Second, it is illegal to purchase stolen property. In this study, it is the latter crime that is the focus of attention, with participants’ attributions of blame with regards to who purchased the stolen camera being assessed.

The filler video was used to create a short break between participants watching the crime video and reading the co-witness statement. The video was an excerpt from a television documentary series about the British coastline and lasted 5 min. It was selected as the content does not in any way overlap with that of the critical incident video, meaning it should not provide any post-event information that could contaminate participants’ memory of the critical incident.

Prior to reading the co-witness statement about the crime, participants were presented with a picture of the co-witness. The picture was either of a 21 year old female or an 82 year old female. Similar photographs were used by Kwong See et al. [35] and these were introduced to emphasise the co-witness’s age. These pictures were selected from a pre-existing database of adult faces designed for research purposes [37]. Both pictures were black and white, had forward-facing profiles showing the women’s head and neck only, and were matched in terms of their perceived familiarity of appearance, mood expressed, picture memorability, and image quality. Both pictures were accompanied by the same fictional co-witness name (Elizabeth Smith) and her age.

There were six versions of the co-witness statement. Each was written from the perspective of the female co-witness. Each statement was identical aside from two features. Depending upon the co-witness age condition, she claimed to be in the pub with either her grandmother or granddaughter. This was done to reinforce the fact that she was a young adult or elderly. There were three versions of each young adult and elderly co-witness statement that varied with respect to who was blamed for purchasing the stolen camera. One-third were control statements where no blame was attributed. In these the co-witness simply said that “*one of the ladies*” purchased the camera. One-third were correct statements with the co-witness correctly stating that “*the lady with the short dark brown hair and white top*” bought the camera. The remaining third were incorrect statements where the co-witness incorrectly stated that “*the lady with the long light brown hair and a grey top*” bought the camera. All other details in the six co-witness statements were correct. The control statements were included as they provide a measure of how accurately participants remembered who was to blame for the crime. If only the correct and incorrect statements were used and participants blamed the same person as the co-witness in these conditions then it would not be possible to determine whether participants were correct in the correct statement conditions as they remembered the incident correctly or because they were engaging in blame conformity.

The 10-item multiple choice questionnaire contained nine general questions regarding the crime video and one critical question regarding who was to blame. The questions were presented one at a time on a PowerPoint display with a choice of four possible answers beneath them. One answer was always ‘*don’t know*’ as real eyewitnesses can withhold answers to questions during police interviews if they are unsure of an answer [38] and this option has been demonstrated to increase their overall accuracy [39–42]. The nine general questions related to different aspects of the video such as the gender of the first group of customers the camera seller approached. None of these questions related to the three individuals who the camera seller approached last. These nine general questions were included to disguise the fact that the study was primarily interested in attributions of blame for the crime. Preliminary analysis of the answers to these nine questions revealed no significant difference in the recall performance of participants in each of the 6 conditions or between those participants who blamed the innocent bystander for the crime after reading an incorrect statement and those who did not (all *p*’s>.05). Consequently, these findings are not discussed any further. For those who are interested, the full anonymised data set from this study is available on the Open Science Framework website (see the following direct link: https://osf.io/ex8p7/?view_only=166afc2821564d37a4341e0dd0340b92). The critical question always appeared 7^th^ and asked ‘*Who bought the camera*?’. The four possible answers were (a) The man with the short brown hair and light brown shirt, (b) The woman with the long light brown hair and grey top, (c) The woman with the short dark brown hair and white top, and (d) don’t know.

The 6-item questionnaire about the co-witness contained a single age manipulation check question and five questions asking participants about their perceptions of the co-witness based on her statement. The age manipulation check question asked participants “*How old was the eyewitness*?” with participants also told “*If you cannot remember her exact age*, *please provide your best guess*". This was included as the study is examining the impact of co-witness age on blame conformity and it is important to establish that participants had noted the co-witness’ age before any claims can be made with regards to how this age impacted upon their performance. The remaining five questions were taken from Brimacombe, Jung, Garrioch, and Allison [43] and required participants to rate their co-witness in terms of how accurate, confident, honest, credible, and competent she seemed. All ratings were made using 7-point Likert scales (1 = not at all; 7 = extremely).

### Procedure

Testing occurred in two sessions that were one week apart. In both sessions the participants were tested within single-person partitioned computer booths that had headphones attached to the computers. All stimuli were presented as part of a PowerPoint presentation. In Session 1, onscreen instructions informed the participants that they would be required to watch two short videos and read some information about one of the videos. The onscreen instructions then asked participants to place their headphones on so that they could hear the audio in the videos and the study commenced. The crime video was presented first. When the crime video finished there was a 5s pause. During this pause there was a warning that the second (filler) video was about to start. After the filler video, onscreen instructions informed participants that they would be shown some information about a co-witness who was in the pub in the first video when the stolen camera was purchased and that they would have 10s to read over this information. The presentation then moved on to reveal a picture of the co-witness, her name, and her age. Participants were then shown a slide containing the co-witness statement and informed they had 2 min to read over it. After the 2 min were over, the slide disappeared. After this, Session 1 ended and participants were asked to return to the laboratory one week later. The decision was made to introduce the co-witness misinformation in Session 1 as surveys suggest most crimes have multiple eyewitnesses and co-witness misinformation can be shared between these co-witnesses prior to them being questioned by the police. The introduction of a one-week delay between sessions is forensically relevant given that there can then be an indefinite time period between an eyewitness observing an incident and recalling it for legal testimony [44].

Session 2 started with onscreen instructions informing participants that they would be required to complete a memory test relating to the first video they saw the previous week. The first of the 10-item multiple choice questions then appeared. Onscreen instructions informed participants that they could navigate through the 10 questions at their own pace using the downward arrow key on the keyboard and that they must select a single answer. Their answers were to be written on a response sheet that was provided by stating whether they were choosing answer *a*, *b*, *c*, or *d*. After this, participants were asked to answer six questions about the co-witness, again navigating through the questions at their own pace using the keyboard and writing their answers on a new response sheet. After this, participants were debriefed, thanked for their time, and asked to refrain from disclosing the aims of the study to other potential participants. The participants were also asked if they were aware of the true aims of the study (in an effort to capture any demand characteristics) and none claimed awareness.

## Results

### Age Manipulation Check

As the study is examining the impact of co-witness age upon blame conformity it is important to establish that participants attended to the co-witness’s age during testing. This was assessed by asking participants to recall her age at the end of the study. Participants were not expected to remember her exact age due to the one week interval between learning it and recalling it. The responses of those in the young adult co-witness condition ranged from 17–32 years of age (*Mean* = 22.94, *Median* = 22.00, *SD* = 3.41) and the responses of those in the elderly co-witness condition ranged from 61–93 years of age (*Mean* = 78.18, *Median* = 81.00, *SD* = 7.24). Participants were, therefore, fairly precise in their responses. This difference was significant, *t*(166) = 55.24, *p* < .001, *d* = 9.76, confirming the age manipulation was successful.

### Blame Conformity Analysis

Two separate 4 x 3 Exact Pearson Chi-Square tests were used to determine if there was an association between who participants blamed for the crime (the innocent man, the innocent woman, the guilty woman, or don’t know) and who the co-witness blamed for the crime (control statement with no blame, correct statement blaming the guilty woman, or incorrect statement blaming the innocent woman). The first test examined participants’ attributions of blame after reading the young adult co-witness statement whereas the second test examined participants’ attributions of blame after reading the elderly co-witness statement. Exact Chi-Square Tests were used as data screening revealed that several contingency table cells had expected counts of less than 5 (for readers unfamiliar with Exact Tests, see [45]). The full range of observed frequencies, in percentages, can be seen in Table 1.

**Table 1:** Percentage of trials on which participants attributed blame for a crime to the innocent man, innocent woman, guilty woman, or responded don’t know after reading a co-witness statement where a young adult or elderly co-witness blamed either nobody (control statements), the innocent woman (incorrect statements), or the guilty woman (correct statements). The percentages shown are within-statement type.

	Statement Type		Participant Blame		
Co-witness Age		*Innocent Man*	*Innocent Woman*	*Guilty Woman*	*Don’t Know*
Young Adult	*Control Statement*	10.70	7.10	75.00	7.10
	*Correct Statement*	3.60	7.10	75.00	14.30
	*Incorrect Statement*	10.70	42.90	35.70	10.70
Elderly Adult	*Control Statement*	0.00	7.10	82.10	10.70
	*Correct Statement*	0.00	3.60	92.90	3.60
	*Incorrect Statement*	7.10	7.10	75.00	10.70

After reading the control statement from the young adult co-witness, 7.10% of participants blamed the innocent woman for the crime whereas 75.00% blamed the guilty woman. An identical trend was observed after participants had read the correct statement from the young adult co-witness. Participants were therefore largely correct in their attributions of blame in these conditions with only a small proportion blaming the man or choosing ‘don’t know’. In contrast, when participants read the incorrect young adult co-witness statement 42.90% blamed the innocent woman whereas 35.70% blamed the guilty woman. This latter result suggests blame conformity occurred. Again, few people blamed the innocent man or selected ‘don’t know’. There was a significant association between who the young adult co-witness blamed for the crime and who participants blamed, *χ* ^*2*^ (6, *n* = 84) = 18.96, *exact p* = .003, *Cramer’s V* = .34. The standardised residuals were examined to determine which cells contained frequencies that differed from those expected if there was no association between who the co-witness blamed and who the participants blamed. To be statistically significant at the .05 level, the standardised residuals need to be +/- 1.96 z-scores. After reading an incorrect young adult co-witness statement blaming the innocent woman for the crime the participants blamed the innocent woman more often than expected (*z* = 2.90). No other cells contained values that significantly differed from the expected frequencies (all *z*’s = <1.96). Combined, these results demonstrate that participants engaged in blame conformity with the incorrect young adult co-witness.

After reading the control statement from the elderly co-witness 7.10% of participants blamed the innocent woman and 82.10% blamed the guilty woman. A similar trend was observed after participants read the correct elderly co-witness statement with 3.60% blaming the innocent woman and 92.90% blaming the guilty woman. No participants in either condition blamed the innocent man and a small percentage selected ‘don’t know’. Crucially, when participants read the incorrect elderly co-witness statement only 7.10% blamed the innocent woman and 75.00% blamed the guilty woman. Again, only a small number of participants blame the man or responded ‘don’t know’. Combined, these results suggest participants were generally correct in their attributions of blame for the crime and that those who read an incorrect elderly co-witness statement did not succumb to blame conformity. In support of this there was no significant association between who the elderly co-witness blamed and who the participants blamed, *χ* ^*2*^ (6, *n* = 84) = 6.09, *exact p* = .46, *Cramer’s V* = .19.

### Co-Witness Questionnaire Analysis

The final analysis examined whether or not there was a difference in how participants perceived the young adult and elderly co-witnesses in terms of their accuracy, confidence, honesty, credibility, and competence. The means and standard deviations for each trait can be seen in Table 2. Participants perceived the young adult and elderly co-witness to be equally confident, *t*(166) = 1.39, *p* = .17, *d* = .21, and equally honest, *t*(166) = .56, *p* = .57, *d* = .08. In contrast, the elderly co-witness was rated as lower than the younger co-witness in terms of accuracy, *t*(166) = 2.96, *p* < .01, *d* = .46, credibility, *t*(166) = 6.41, *p* < .01, *d* = .98, and competence, *t*(166) = 7.26, *p* < .01, *d* = 1.12.

**Table 2:** Participants’ perceptions of the young adult and elderly co-witness’s accuracy, confidence, honesty, credibility, and competence. Each trait was scored on a scale of 1–7 (1 = not at all; 7 = extremely).

Co-Witness Trait	*Co-Witness Age*	
	*Young Adult*	*Elderly Adult*
*Accuracy*	5.75 (.96)	5.27 (1.12)
*Confidence*	5.68 (1.02)	5.89 (0.98)
*Honesty*	5.86 (0.79)	5.94 (1.09)
*Credibility*	5.65 (0.96)	4.55 (1.25)
*Competence*	5.86 (0.96)	4.57 (1.31)

## Discussion

This study examined whether or not exposing eyewitnesses to a co-witness statement that incorrectly suggests an innocent bystander was the perpetrator of a crime can increase the likelihood of these eyewitnesses subsequently blaming the innocent bystander for the crime. It also examined whether or not the perceived age of the co-witness who wrote this statement influences this effect. To assess this, participant eyewitnesses watched a video of a crime featuring a perpetrator and an innocent bystander. They then read a statement from a young adult or elderly female co-witness that blamed nobody for the crime, correctly blamed the perpetrator for the crime, or incorrectly blamed the innocent bystander for the crime. When subsequently asked who committed the crime, just over 40% of participants who read the young adult co-witness’s incorrect statement also blamed the innocent bystander. Less than 8% of participants who read the elderly co-witness’s incorrect statement blamed the innocent bystander. Similarly, less than 8% of participants in each of the remaining four conditions blamed the innocent bystander. Exposing an eyewitness to post-event misinformation from a young adult, but not elderly, co-witness who incorrectly blames an innocent bystander for a crime can therefore increase the likelihood of the eyewitness subsequently blaming the innocent bystander for the crime. When asked to rate their co-witness on a number of traits, participants rated the elderly co-witness as less accurate, less competent, and less credible. This occurred despite her statement being identical to that of the young adult co-witness. This suggests that eyewitnesses are less likely to be influenced by an elderly co-witness’s incorrect statement as they perceived her to be a less reliable source of information.

This study extends our understanding of the conditions under which innocent bystanders can be blamed for a crime. It had previously been demonstrated that if eyewitnesses are exposed to a crime involving a perpetrator and an innocent bystander and then administered with a lineup in which both individuals appear then they can engage in unconscious transference whereby they spontaneously mistakenly identify the innocent bystander as the perpetrator [6]. It had also been demonstrated that eyewitnesses are at greater risk of committing such an error if, prior to completing the lineup, they encounter post-event misinformation in a leading question that suggests the innocent bystander was the perpetrator [13]. The current study differed from this earlier work in several ways but two key differences were that participant eyewitnesses were exposed to post-event misinformation via a co-witness statement that was supposedly written by a young adult or elderly adult (and not a leading question) and participants were asked who committed the crime (instead of completing a lineup). This is the first study to demonstrate that participant eyewitnesses are at increased risk of blaming an innocent bystander for a crime after exposure to post-event misinformation from a young adult, but not elderly, co-witness and that this can occur when memory of the perpetrator is assessed in this way.

Prior to this study it was known that if an eyewitness is exposed to a young adult co-witness’s incorrect description of an event, whether directly during a co-witness discussion or indirectly through a third party, then the eyewitness can succumb to memory conformity and incorporate this misinformation into their own descriptions of the event [21–32, 34, 35, 46]. It was unknown whether or not exposure to post-event misinformation from a young adult co-witness who incorrectly blames an innocent bystander for a crime could increase the likelihood of eyewitnesses also blaming the innocent bystander for the crime. There was, however, evidence to suggest this may be possible. Thorley and Rushton-Woods [33] presented participants with a video of an accident involving two men, both of whom were equally responsible for its occurrence, and then had their participants read a co-witness statement written by a young female that incorrectly blamed one of the two men for the accident. When subsequently asked who was responsible for the accident nearly 40% of eyewitnesses exposed to the incorrect statement also blamed the same man. Thorley and Rushton-Woods referred to this more specific form of memory conformity as blame conformity. Despite this finding, it is reasonable to speculate that eyewitnesses may be more inclined to engage in blame conformity when the to-be-remembered event is an accident involving two people who are both equally responsible for it, compared to when it is a crime where one single identifiable person was responsible. Eyewitnesses did, however, also engage in blame conformity with a young adult co-witness who incorrectly blamed an innocent bystander for a crime. This study therefore demonstrates a novel way in which co-witness misinformation can lead to inaccurate eyewitness testimony.

Prior to this study it was also known that individuals are less likely to succumb to memory conformity when misinformation derives from elderly person than a young adult [34–35]. It was, however, unknown whether or not a similar pattern of results would be observed in relation to blame conformity. Given that blame conformity is a more specific form of memory conformity it seemed likely this would occur. As anticipated, a similar pattern of results was observed. Indeed, there was no evidence of blame conformity at all after participants read the elderly adult co-witness’s incorrect statement as the number of participants who blamed the innocent bystander in this condition was equivalent to the number of participants who did so after reading her correct statement or the statement in which she blamed no one for the crime.

Kwong See et al. [35] found that participants are less likely to succumb to memory conformity when presented with misinformation deriving from an elderly co-witness than a young adult co-witness as they perceive the elderly co-witness to be less competent. Whilst this was also observed here, it was also found that participants rated the elderly participant as less credible and less accurate than the younger co-witness despite their eyewitness statements being similar. These findings are consistent with the stereotypical belief that aging is accompanied by a decline in memory [47–48]. Ross, Dunning, Toglia, and Ceci [49] demonstrated that such beliefs can influence perceptions of eyewitnesses with undergraduate participants in their study indicating that they expected a 74 year old eyewitness to have a less accurate memory than a 21 year old eyewitness. Indeed, 34% of their sample believed that it was possible for a person to become too old to be a competent witness.

Wright et al. [17] suggest that individuals can succumb to memory conformity for one of three reasons. First, they may succumb as a result of normative influence whereby they knowingly report errant information suggested by others in order to be liked and accepted by them. Second, they may succumb as a result of informational influence whereby they are uncertain of the truth and consequently report the suggested errant information out of a desire to be correct. Third, they may succumb as they have formed an episodic memory of the errant information. Individual participants within the same study may succumb to memory conformity for different reasons (e.g., [50]). It is beyond the scope of this study to determine why individual participants succumbed but it seems unlikely they did so as a result of normative influence as they did not work alongside a real co-witness who they could be liked and accepted by. Instead, the overall pattern of results suggests the blame conformity in this study was driven informational influence. This explanation is favoured as participants were more likely to succumb to blame conformity when the source of the misinformation was perceived as competent, credible, and accurate. It is speculated that participants who read an incorrect statement about who was to blame had doubt put in their minds with regards to the truth. Those who read the inaccurate statement from the young adult co-witness may have felt inclined to believe this source but those in the elderly co-witness condition may have felt the source was unreliable and relied upon their own accurate memory of the crime instead. It is, however, not possible to rule out that some participants who engaged in blame conformity may have generated false memories of the crime and genuinely believed the innocent bystander was the perpetrator.

An alternative explanation for the age effects observed in this study is that the specific pictures used of the young adult and elderly co-witness influenced participant’s attributions of blame. The pictures were matched on a range of qualities including their perceived familiarity of appearance, mood expressed, picture memorability, and image quality (see [37]). It may be the case, however, that the younger adult in the photograph looked like a more ‘credible’, ‘competent’, or ‘accurate’ person irrespective of her age and participants were more likely to engage in blame conformity with her for this reason. This alternative explanation cannot be completely ruled out but it does seem unlikely. The findings from this study are consistent with those of previous studies that also used photographs of a young adult or elderly co-witness [35] or had participants work with a real young adult or elderly co-participant [34]. Co-witness age differences in memory conformity therefore seem to be robust and not an artefact of the physical appearance of the specific people used as the young adult and elderly co-witnesses/co-participants.

These findings from this study have applied relevance. Most crimes have multiple eyewitnesses [14–15] and post-event misinformation deriving from one co-witness can contaminate the legal testimony of another (for examples see [17–19]). If an eyewitness is exposed to post-event co-witness misinformation suggesting an innocent bystander was the perpetrator of a crime then this eyewitness could subsequently report that this innocent person was to blame when questioned by the police. This could then waste police resources in several ways. If the innocent bystander was no longer present at the crime scene then the police would have to devote resources to searching for this individual. If the innocent bystander was later found then the police could waste time asking the innocent bystander irrelevant questions about his or her role in the crime. If the innocent bystander was subsequently charged with the crime then this could lead to a miscarriage of justice. Following concerns over the negative impact that exposure to co-witness misinformation can have on the reliability of eyewitness testimony, countries such as the United States [51, 52] and the United Kingdom [53] have created guidelines recommending that police officers keep co-witnesses separate and that they establish whether or not they have spoken to each other in order to determine whether their testimonies can be classed as truly independent evidence or not. The findings from the present study emphasise the importance of this.

One limitation to the ecological validity of the current findings is the way in which memory of the perpetrator was assessed. Here, participants answered a multiple choice recognition test question that asked them who committed the crime and the response options were the female perpetrator, a male innocent bystander, a female innocent bystander, or ‘don’t know’. This was done to ensure all participants were questioned about the perpetrator’s identity in a similar way. In real police interviews eyewitnesses are unlikely to be given multiple choice questions about a crime. Instead, police interviewers in many countries (e.g., the United States, see [51, 52]; the United Kingdom, see [54]) are encouraged to assess eyewitness memory of an incident via free recall before asking open-ended cued-recall questions. As discussed by Koriat and Goldsmith (see [40–41]), free recall produces largely accurate recollection as it requires self-guided retrieval, meaning that eyewitnesses typically only report information they are confident about. In contrast, multiple choice tests utilise externally-guided retrieval where the interviewer dictates what information is important and thus what information the eyewitness reports. This leads individuals to report information which they may not necessarily choose to report because they are unsure of it and can result in increased errors and confabulations. It is therefore possible that the use of a multiple choice question in the current study to assess memory of who committed the crime, when one option was the innocent bystander, inflated blame conformity effects in the incorrect statement condition. To control for this the current study allowed participants to respond ‘*don’t know*’ to all questions. This is forensically important as real eyewitnesses can withhold answers to questions during interviews if they are unsure of an answer [38]. It is also methodologically important as providing this option during multiple choice memory tests has been found to increase eyewitness memory accuracy (e.g., [39–42]). Thus, whilst the ecological validity of this study may have been lowered by the use of a multiple choice question to assess memory of the perpetrator it is unlikely that the use of this question increased susceptibility to blame conformity.

There is an important outstanding issue that requires consideration in future research. It is possible that degree of similarity between an innocent bystander and a perpetrator may influence the degree to which eyewitnesses engage in blame conformity. Research from the unconscious transference literature suggests that eyewitnesses are more likely to spontaneously blame an innocent bystander for a crime if they are similar in appearance to the perpetrator [55]. In the present study (and in [33]) blame for an incident was shifted between two individuals who were similar in terms of their gender, race, and age. It may have been the case that these ethnographic similarities facilitated blame conformity in this study as participants felt they may have simply misremembered which young white female committed the crime. The perpetrator and innocent bystander did differ in several ways (e.g., hair colour, hair length, colour of clothing). Several other salient differences, such as their gender or race, could have also existed between them. It would be of interest to determine whether or not blame conformity still occurs when such salient differences do exist.
